# Isolated Capitulum-Trapezoid Coalition: An Unusual Cause of Wrist Pain

**DOI:** 10.7759/cureus.49828

**Published:** 2023-12-02

**Authors:** Amjaad W Almubarzi, Mashael A Alhussain, Nurah A Alkhteeb, Waad D AlOtaibi, Alwaleed M Mohammed

**Affiliations:** 1 General Practice, King Faisal University, Hofuf, SAU; 2 Orthopedic Surgery, Dallah Hospital, Riyadh, SAU

**Keywords:** trapezoid, capitulum, syndesmosis, carpal coalition, chronic wrist pain

## Abstract

Chronic wrist pain presents a diagnostic challenge, demanding a comprehensive understanding of its multifactorial etiology. This case report focuses on wrist coalition, an infrequent orthopedic condition characterized by abnormal articulations between carpal bones, often associated with prolonged wrist discomfort. A 45-year-old man with a two-year history of persistent left wrist pain, with no previous history of trauma or systemic illness, had a localized tenderness in the left carpal region with a restricted range of motion, and diminished grip strength on physical examination. Initial investigations, including autoimmune profiles and plain radiographs, were inconclusive. Magnetic resonance imaging ultimately identified a coalition between the capitulum and the trapezoid. A multidisciplinary team recommended a conservative approach, resulting in a positive response and symptomatic improvement during follow-up. This case report contributes valuable insights to the limited literature on isolated capitulum and trapezoid coalition, highlighting the significance of considering such rare orthopedic entities in the comprehensive evaluation of chronic wrist pain.

## Introduction

The wrist, a complex joint comprising multiple bones, ligaments, and tendons, is crucial for a myriad of hand movements and functions. Wrist pain is a common complaint encountered in clinical practice, often arising from a myriad of etiologies ranging from traumatic injuries to degenerative conditions [1]. Wrist coalition, typically an embryological anomaly, occurs when the normal segmentation of carpal bones during development is disrupted, leading to fusion and subsequent alterations in joint mechanics [2]. While the literature extensively covers common wrist pathologies, symptomatic coalition-related cases are infrequently reported [1,2]. Understanding and recognizing these rare conditions is crucial for accurate diagnosis and tailored treatment.

The capitulum and trapezoid coalition, as observed in our case, add a distinctive dimension to the spectrum of wrist pathologies. Notably, the challenge lies in diagnosing such anomalies, given their subtle presentation on routine imaging. This case underscores the significance of magnetic resonance imaging in unraveling complex wrist pathologies that may elude conventional diagnostic approaches.

## Case presentation

A 45-year-old right-handed male presented with a two-year history of persistent left wrist pain. The pain manifested insidiously, gradually worsening over time, and was exacerbated by movement, without any associated history of trauma or systemic illness. Occupational history revealed engagement in repetitive manual tasks. The patient’s medical history yielded no remarkable findings, and the social and family history was unremarkable.

Upon examination, localized tenderness was noted over the dorsal aspect of the left wrist, accompanied by a restricted range of motion, particularly in extension and radial deviation. Grip strength was notably diminished, and no signs of systemic inflammation were observed.

Routine blood work, encompassing a complete blood count, erythrocyte sedimentation rate, and C-reactive protein, returned normal results (Table 1). Additionally, rheumatologic markers were negative, and the plain radiograph displayed no significant abnormalities.

**Table 1:** Laboratory test results.

Test	Result	Reference range
Complete blood count (CBC)		
White blood cell count (WBC)	7.2 × 10^3^/μL	4.0 - 11.0 × 10^3^/μL
Red blood cell count (RBC)	5.0 × 10^6^/μL	4.5 - 5.5 × 10^6^/μL
Hemoglobin (Hb)	15.0 g/dL	13.5 - 17.5 g/dL
Hematocrit (Hct)	0.452	38.3 - 48.6%
Mean corpuscular volume (MCV)	90 fL	80 - 100 fL
Mean corpuscular hemoglobin (MCH)	30 pg	27 - 33 pg
Mean corpuscular hemoglobin concentration (MCHC)	34 g/dL	32 - 36 g/dL
Platelet count	300 × 10^3^/μL	150 - 450 × 10^3^/μL
Inflammatory markers		
C-reactive protein (CRP)	0.2 mg/dL	0.0 - 0.5 mg/dL
Erythrocyte sedimentation rate (ESR)	8 mm/hour	0 - 15 mm/hour
Rheumatologic markers		
Rheumatoid factor (RF)	10 IU/mL	0 - 14 IU/mL
Antinuclear antibodies (ANA)	Negative	Negative
Cyclic citrullinated peptide (CCP)	15 U/mL	<20 U/mL
Anti-neutrophil cytoplasmic antibodies (ANCA)	Negative	Negative

**Figure 1:**
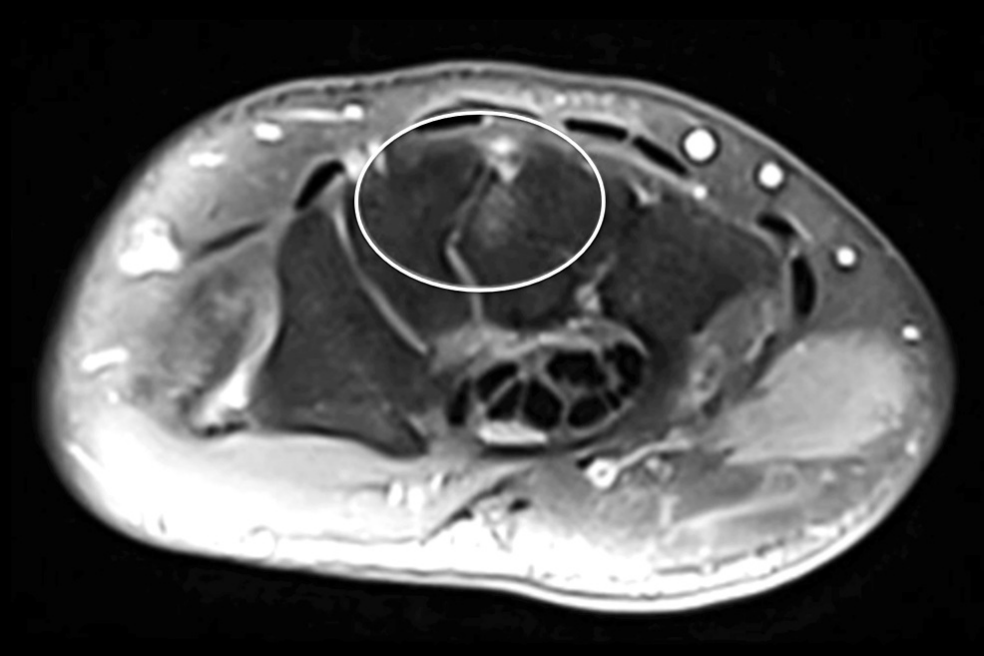
PD-weighted axial MRI image of the left wrist showing the abnormal fibrous coalition (encircled) between the capitulum and the trapezoid. PD: proton density; MRI: magnetic resonance imaging.

**Figure 2:**
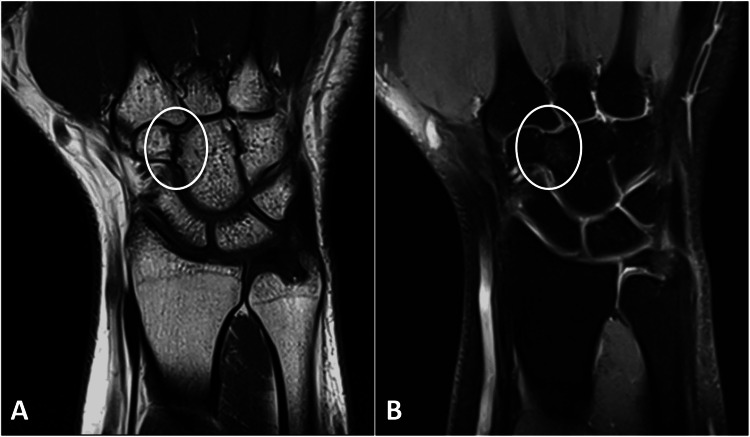
Coronal MRI images of the left wrist are displayed, featuring T1-weighted (A) and PD-weighted (B) images. The illustrations depict the abnormal fibrous coalition (encircled) between the capitulum and the trapezoid, with minimal bone edema noted. PD: proton density; MRI: magnetic resonance imaging.

The differential diagnosis initially considered ligamentous injury, inflammatory arthropathy, and avascular necrosis. However, further investigation through magnetic resonance imaging revealed an anomalous articulation between the capitulum and the trapezoid, consistent with a coalition (Figures 1, 2).

A multidisciplinary team, comprising orthopedic surgery, rheumatology, radiology, and physiotherapy, recommended a conservative approach involving analgesics with paracetamol and non-steroidal anti-inflammatory drugs, splinting, and physiotherapy. Surgical intervention would only be contemplated if symptoms persisted or worsened. Subsequent follow-up appointments revealed a positive response to conservative management, with partial resolution of pain and a decreased analgesic requirement. Furthermore, the physical examination showed an improved range of motion and grip strength. The patient’s progress continues to be closely monitored to ensure sustained improvement and address any emerging concerns.

## Discussion

Chronic wrist pain remains a complex clinical presentation, often necessitating a thorough exploration of diverse etiologies [1]. Our case emphasizes the crucial role of careful evaluation in identifying unusual causes of persistent wrist pain. Conventionally, ligamentous injuries, inflammatory arthropathies, and avascular necrosis are common etiologies for wrist pain [1]. However, our case prompts consideration of rare structural anomalies, particularly carpal coalitions, when plain radiographs were unremarkable.

Carpal coalitions, defined as abnormal unions between adjacent carpal bones, present a diagnostic conundrum [2]. While previous literature has extensively documented common wrist pathologies, reports on carpal coalitions, particularly isolated capitulum and trapezoid coalitions, are notably scarce [3,4]. The wrist’s anatomy, characterized by a complex network of ligaments and articulations, makes it susceptible to subtle aberrations that can manifest as chronic pain.

Understanding the diverse types of carpal coalitions is imperative. Coalitions can manifest between various carpal bones, including the lunate and triquetrum, hamate and capitate, or, as in our case, the capitate and trapezoid [2]. The classification of carpal coalitions encompasses syndesmotic and synostotic types. Syndesmotic coalitions involve fibrous connections between bones, while synostotic coalitions signify bony unions [2]. Our case, revealing synostosis between the capitulum and trapezoid, exemplifies the latter. The distinction between these types holds clinical relevance, influencing the prognosis and therapeutic approach. Syndesmotic coalitions may allow for greater mobility and response to conservative measures, while synostotic coalitions often necessitate more judicious management, considering the potential impact on joint function and adjacent soft tissues [5].

In our patient’s case, the decision for conservative management aligns with existing evidence supporting non-surgical approaches for selected instances of carpal coalition. The multidisciplinary team’s recommendation of analgesics, splinting, and physical therapy aims to alleviate symptoms and improve wrist function. However, the potential for surgical intervention remains a consideration, particularly if symptoms persist or worsen.

## Conclusions

In conclusion, chronic wrist pain demands a comprehensive diagnostic approach that considers not only common pathologies but also rare structural anomalies like carpal coalitions. The complex anatomy of the wrist, combined with the varied types of coalitions and their potential clinical implications, requires clinical judgment and advanced imaging for accurate diagnosis. Our case report contributes to the limited literature on isolated capitulum and trapezoid coalition. As our understanding of these entities evolves, this case adds valuable insights to the broader discourse on wrist pain and carpal coalitions, guiding clinicians in navigating the complexities of diagnosis and management.

## References

[1] Ferguson R, Riley ND, Wijendra A, Thurley N, Carr AJ, Bjf D (2019). Wrist pain: a systematic review of prevalence and risk factors- what is the role of occupation and activity?. BMC Musculoskelet Disord.

[2] Defazio MV, Cousins BJ, Miversuski RA Jr, Cardoso R (2013). Carpal coalition: a review of current knowledge and report of a single institution's experience with asymptomatic intercarpal fusion. Hand (N Y).

[3] Christ AB, Maertens AS, Weiland AJ (2016). Bilateral complete osseous coalition of the capitate and trapezoid. J Wrist Surg.

[4] Peters S, Colaris JW (2011). Carpal coalition: symptomatic incomplete bony coalition of the capitate and trapezoid--case report. J Hand Surg Am.

[5] Gottschalk MB, Danilevich M, Gottschalk HP (2016). Carpal coalitions and metacarpal synostoses: a review. Hand (N Y).

